# Oxidative Stress Activates the Transcription Factors FoxO 1a and FoxO 3a in the Hippocampus of Rats Exposed to Low Doses of Ozone

**DOI:** 10.1155/2014/805764

**Published:** 2014-05-22

**Authors:** Nancy P. Gómez-Crisóstomo, Erika Rodríguez Martínez, Selva Rivas-Arancibia

**Affiliations:** Departamento de Fisiología, Facultad de Medicina, Universidad Nacional Autónoma de México, Apartado Postal No. 70250, 04510 Delegación Coyoacán, DF, Mexico

## Abstract

The exposure to low doses of ozone induces an oxidative stress state, which is involved in neurodegenerative diseases. Forkhead box O (FoxO) family of transcription factors are activated by oxidative signals and regulate cell proliferation and resistance to oxidative stress. Our aim was to study the effect of chronic exposure to ozone on the activation of FoxO 1a and FoxO 3a in the hippocampus of rats. Male Wistar rats were divided into six groups and exposed to 0.25 ppm of ozone for 0, 7, 15, 30, 60, and 90 days. After treatment, the groups were processed for western blotting and immunohistochemistry against FoxO 3a, Mn SOD, cyclin D2, FoxO 1a, and active caspase 3. We found that exposure to ozone increased the activation of FoxO 3a at 30 and 60 days and expression of Mn SOD at all treatment times. Additionally, increases in cyclin D2 from 7 to 90 days; FoxO 1a at 15, 30, and 60 days; and activate caspase 3 from 30 to 60 days of exposure were noted. The results indicate that ozone alters regulatory pathways related to both the antioxidant system and the cell cycle, inducing neuronal reentry into the cell cycle and apoptotic death.

## 1. Introduction

Neurodegenerative diseases, such as Alzheimer's, Parkinson's, and Creutzfeldt-Jakob disease, are a set of disorders that affect specific groups of neurons, inducing chronic, progressive, and irreversible dysfunction [1]. These pathologies share chronic features, that is, abnormal protein aggregation, excitotoxicity, neuroinflammation, and oxidative stress [2]. Several conditions have been associated with redox imbalance, and aging is one of the most important risk factors for the development of neurodegenerative diseases [3]. Many evidences suggest that the central nervous system (CNS) is highly sensitive to oxidative stress, because of its high content of unsaturated phospholipids, its high metabolic rate, and low content of some antioxidant enzymes, such as catalase [4, 5], the hippocampus, substantia nigra, and striatum being the most sensitive structures [6]. Additionally, exposure to air pollution may contribute to the development of neurodegenerative diseases [7]. Increasing exposure to environmental contaminants has attracted the attention of researchers because it plays an important role in the risk factors associated with mortality and accounts for 2.5% of all deaths in developing countries [8]. The effect of exposure to air pollution has been extensively studied in the respiratory and cardiovascular systems; however, its impact on the central nervous system (CNS) is poorly understood.

The forkhead box (Fox) family of transcription factors regulates a myriad of cellular functions. These transcription factors are involved in the regulation of metabolism, cell proliferation, resistance to stress, immune system regulation, and apoptosis [9]. Activation of these transcription factors can be regulated by growth factors such as insulin-like growth factor (IGF), which promotes FoxO phosphorylation at its C-terminal end via protein kinase B (AKT/PKB), resulting in nuclear exclusion and a subsequent loss of transcriptional activity [10]. Reactive oxygen species (ROS) may also induce the activation of these transcription factors through posttranslational modifications such as phosphorylation via the JNK pathway [11], methylation, and ubiquitination [12]. Therefore, the FoxO family is considered to play a central role in redox signaling [13]. These transcription factors have a highly conserved DNA binding domain (approximately 100 amino acids) and are tissue specific; thus, the activation pathway determines the functional effects of FoxOs in a particular cell [14]. FoxO 1a participates in the regulation of gluconeogenesis and glycogenolysis by controlling the expression of glucose-6-phosphatase and phosphoenolpyruvate carboxykinase, and it also participates in cell cycle regulation by downregulating the transcription of cyclin D [15, 16]. In addition FoxO 1a is involved in resistance to oxidative stress via the upregulation of antioxidant enzymes such as catalase and Mn SOD among FoxO 3a [4, 17]; FoxO 3a is also linked to apoptotic processes by modulating the expression of proapoptotic (e.g., Bim and PUMA) and antiapoptotic (FLIP) proteins [10].

Our group has developed a murine model of neurodegeneration by chronic exposure to low doses of ozone, a major component of air pollution. With this model, it was shown that chronic exposure to ozone generates a state of oxidative stress that induces damage in rat brains [14]. Previously, we reported an increase in oxidized lipids and proteins in addition to a loss of the brain repair processes in the hippocampi and substantia nigra of the animals throughout the treatment [6, 18].

The aim of this work was to study the effect of chronic exposure to ozone on cell signaling related to oxidative stress response, cell cycle, neuronal repair, and apoptosis by analyzing the expression and phosphorylation of FoxO transcription factors, FoxO 3a and 1a, as well as the expression of Mn SOD, cyclin D2, and caspase 3 in the hippocampus of rats.

## 2. Experimental Procedures

### 2.1. Exposure to Ozone

A total of 72 male Wistar rats (250–300 g) were maintained individually in acrylic boxes with free access to water and food. The animals were randomly divided into six groups: (1) control (exposure to ozone-free air); (2) exposure to ozone for 7 days; (3) exposure to ozone for 15 days; (4) exposure to ozone for 30 days; (5) exposure to ozone for 60 days; and (6) exposure to ozone for 90 days (*n* = 12 per group). No differences between the control groups were detected at any time during ambient air exposure; therefore, we chose the 30-day air-exposure group as a control. Exposure to ozone was performed according to the method previously described by Rivas-Arancibia et al. [18]. Briefly, rats were placed in a transparent acrylic chamber that was then sealed, and the rats were exposed to ozone (0.25 ppm) for 4 hours daily over the period of time indicated for each treatment (7, 15, 30, 60, or 90 days). The ozone concentration in the chamber was monitored throughout the experiment with an ozone monitor (PCI Ozone and Control Systems, West Caldwell, NJ). After the exposure, the rats were deposited in individual boxes. Control rats were subjected to the same protocol with exposure to ambient airflow. All experiments were conducted according to the guidelines of the National Institutes of Health and the Mexican Official Standard (NOM-062-ZOO 1999).

### 2.2. Western Blot

Two hours after the end of treatment, the animals were deeply anesthetized with pentobarbital sodium (50 mg/kg) and sacrificed. The hippocampi were dissected, homogenates were prepared in buffer, and protein was quantified by the Bradford method. The samples were mixed with 5X loading buffer (0.5 M Tris [pH 8.5], 10% SDS, 30% glycerol, 0.1% bromophenol blue, and 100 mM dithiothreitol) and boiled for 10 minutes. Subsequently, the proteins were separated by 10% denaturing polyacrylamide gel electrophoresis and transferred to a polyvinylidene fluoride membrane (PVDF; Immobilon-P Transfer Membranes, Millipore Corporation, Billerica, MA, USA). The membranes were blocked for 2 hours at room temperature with 5% skim milk in Tris phosphate buffer/Tween 20 (0.1%). After washing, the membranes were incubated overnight with primary antibodies (anti-rabbit FoxO 1a, anti-rabbit FoxO 3a, anti-rabbit GAPDH, anti-mouse cyclin D2, and anti-rabbit Mn SOD) at 1 : 1000 dilutions. Subsequently, the membranes were washed and incubated for 2 hours with the corresponding HRP-conjugated secondary antibody (anti-rabbit IgG or anti-mouse IgG) diluted 1 : 10,000. The chemiluminescence signal was detected with Immobilon Western Chemiluminescent HRP Substrate (Millipore Corporation, Billerica, MA, USA).

### 2.3. Immunohistochemistry

Two hours after the last exposure to ozone, the rats (*n* = 6 per group) were deeply anesthetized and perfused with 4% paraformaldehyde. The brains were dissected and placed in the same fixative solution for 24 hours at 4°C. Subsequently, conventional histological techniques were performed to obtain tissue embedded in paraffin. Sagittal sections (5 *μ*m thickness) were cut, and those that contained the hippocampus were used for immunohistochemistry. The slices were deparaffinized with xylene and rehydrated; subsequently, the slices were washed with phosphate-buffered saline (PBS; 50 mM sodium phosphate, 0.15 M sodium chloride, pH 7.4) and incubated with 2% fatty acid-free bovine serum albumin (fraction V; MP Biomedical, LLC., USA) for 30 minutes to prevent nonspecific binding. Subsequently, the samples were permeated with 0.2% Triton X100 in PBS for 10 minutes before overnight incubation with the primary antibody at 4°C. Lastly, the preparations were incubated with the corresponding FITC-conjugated secondary antibody. Additionally, the slices were counterstained with Vectashield with DAPI (Vector Labs, CA, USA) for nuclei staining. Confocal micrographs were obtained on a Leica microscope (Leica TCS-SP5).

### 2.4. Statistical Analysis

The data are presented as the mean ± SD and were analyzed using one-way ANOVA followed by Dunnett post hoc to evaluate the statistical significance. A *P* value < 0.05 was considered as a threshold for statistical significance.

## 3. Results

### 3.1. Activation of FoxO 3a

**Figure 1:**
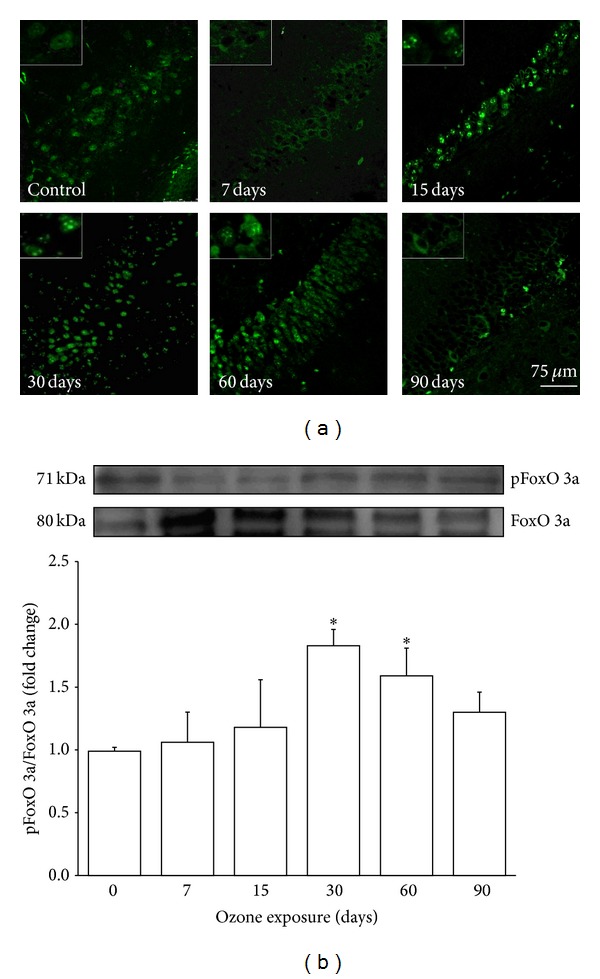
Effect of ozone exposure on pFoxO 3a in the rat hippocampus. (a) The micrographs show cells that are positive for phosphorylated FoxO 3a (pFoxO 3a) (green) in the dentate gyrus (DG) of rats exposed to ozone-free air (control) or 7, 15, 30, 60, and 90 days of ozone. The images show a progressive increase in the immunoreactivity against phosphorylated FoxO 3a from 15 days to 60 days of exposure to ozone. pFoxO 3a shows nuclear localization from 15 days to 60 days of ozone exposure and at 90 days is located in the cytoplasm. Insets are a zoom from selected areas in the same picture. (b) A representative western blot shows the contents of total FoxO 3a and pFoxO 3a in the homogenated hippocampi of rats exposed to ozone for different durations. The normalized graph shows densitometry values presented as the mean ± SD (^∗^ *P* < 0.05) (*n* = 6).

Activation was evaluated by identifying the phosphorylated form of FoxO 3a (pFoxO 3a). We detected a gradual increase in the immunoreactivity in the dentate gyrus (DG) of the rat hippocampus. This increase was evident from the 15th to the 60th day of exposure to ozone (Figure 1(a)). The photomicrographs illustrate that this protein is mainly localized in the nucleus of the DG cells (Figure 1(a)). Moreover, western blot analysis showed a significant increase in the pFoxO 3a protein after 30 days and 60 (^∗^ *P* < 0.05) days of exposure to ozone (relative to the control). There was also an increase with respect to the control at 90 days of exposure; however, this increase was not statistically significant.

### 3.2. Mn SOD Protein Expression

**Figure 2:**
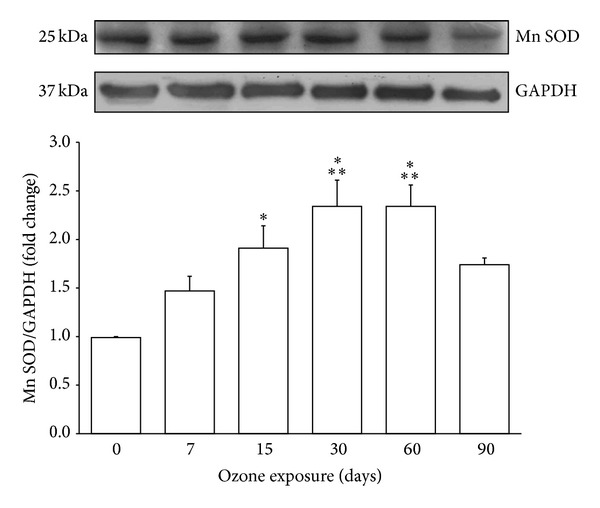
Effect of ozone treatment on Mn SOD in the hippocampus. A representative western blot and densitometric analysis of six independent experiments revealed a gradual increase in Mn SOD from 15 days to 60 days of exposure to ozone (^∗^ *P* < 0.05 with respect to control) and differences at 30 and 60 days compared with 7 days of treatment (^∗∗^ *P* < 0.05). The graph shows mean values ± SD. GAPDH was used as a loading control.

Mn SOD protein expression was examined by western blot. This protein showed a gradual, statistically significant increase as early as 15 days and as late as 60 days (^∗^ *P* < 0.05) of exposure to ozone compared with the control and increase at 30 and 60 days compared with 7 days (^∗∗^ *P* < 0.05) (Figure 2).

### 3.3. Analysis of Cyclin D2

**Figure 3:**
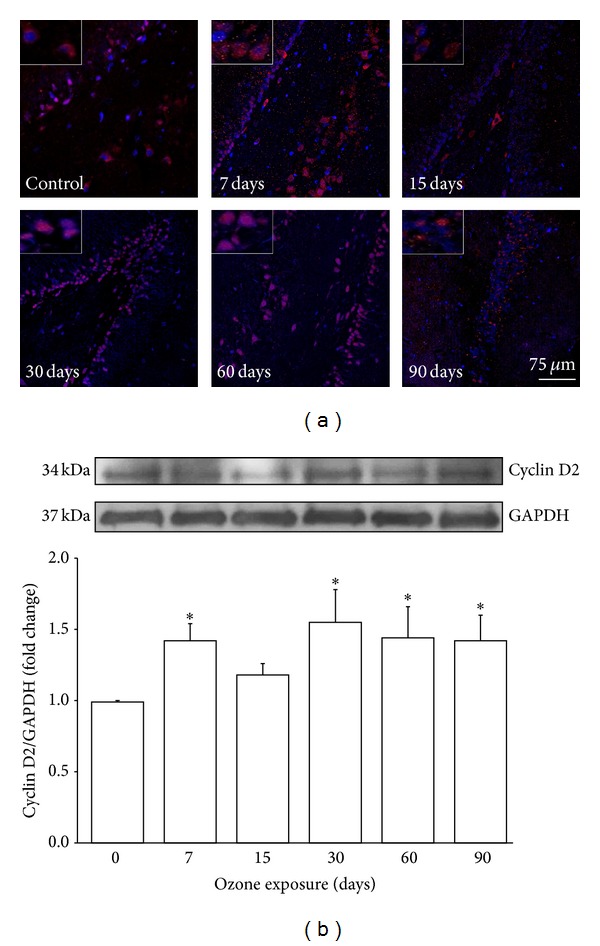
Effect of ozone exposure on cyclin D2. (a) Photomicrographs of cyclin D2-positive cells (red) in the dentate gyrus (DG) of rat hippocampi exposed to ozone for different periods of time (7, 15, 30, 60, and 90 days) or exposed to ozone-free air (control). An increase from 7 days to 90 days of ozone exposure was observed. This protein shows nuclear localization from 30 days to 90 days of ozone exposure. Insets are a zoom from selected areas in the same picture. (b) A representative western blot illustrating changes throughout the treatment. The graph shows densitometry values presented as the mean ± SD (^∗^ *P* < 0.05) (*n* = 6).

As result of ozone exposure, a gradual increase in cyclin D2-immunoreactive cells was observed; after 30 days of treatment, an increase in cyclin D2 nuclear translocation in DG cells of rat hippocampi was also observed (Figure 3(a)). Western blot analysis showed a statistically significant increase in cyclin D2 protein at 7, 30, 60, and 90 (^∗^ *P* < 0.05) days of ozone treatment compared with the control group (Figure 3(b)).

### 3.4. Activation of FoxO 1a

**Figure 4:**
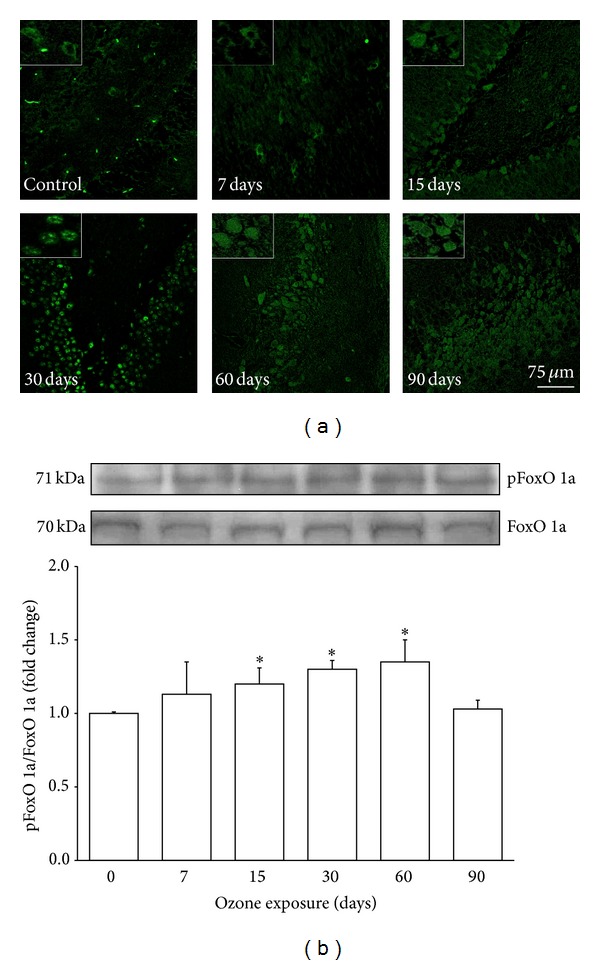
Effect of ozone exposure on pFoxO 1a immunoreactivity in the rat hippocampus. (a) The photomicrographs show pFoxO 1a-positive cells (green) in the dentate gyrus (DG) region. Rats were exposed to ozone-free air (control) or 7, 15, 30, 60, and 90 days with ozone. A progressive increase can be observed in the immunoreactivity against pFoxO 1a from 15 days to 60 days of ozone exposure. Insets are a zoom from selected areas in the same picture. (b) A representative western blot shows the content of the total FoxO 1a and pFoxO protein in the hippocampi of rats exposed to ozone for different times. The graph shows densitometry values presented as the mean ± SD (^∗^ *P* < 0.05).

Cells that were immunoreactive to FoxO 1a were observed in the hippocampi of rats after all ozone treatments, and immunoreactive cells showed a gradual increase with increasing ozone exposure time (Figure 4(a)). Western blot analysis showed significant increases in FoxO 1a protein at 15, 30, and 60 (^∗^ *P* < 0.05) days of exposure to ozone compared with the control (Figure 4(b)).

### 3.5. Analysis of Activated Caspase 3

**Figure 5:**
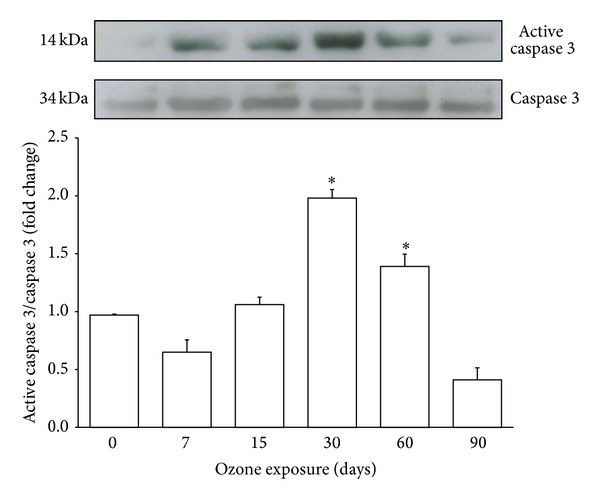
Effect of ozone exposure on the activation of caspase 3 in the hippocampus. A representative western blot and densitometric analysis of six independent experiments showing an increase in activated caspase 3 from 30 days to 60 days and a decrease at 90 days of exposure to ozone with respect to the control. The graph shows mean values ± SD (^∗^ *P* < 0.05).

Western blot analysis of caspase 3 showed a statistically significant increase in the amount of the active enzyme from 30 days to 60 days of ozone exposure and a decrease at 90 days (^∗^ *P* < 0.05) of ozone exposure with respect to total caspase 3 (Figure 5).

## 4. Discussion

Based on the present results, we have demonstrated increased activation of FoxO 3a via the identification of its phosphorylated form (pFoxO 3a; Figure 1). According to a previous report by Kops et al. [19], an increase in pFoxO 3a may be associated with an increased amount of Mn SOD, as shown in the present study (Figure 2). This finding suggests that FoxO 3a has a regulatory role in response to increasing oxidative stress generated by exposure to low doses of ozone. Previous experiments by our group have shown that the activity of this enzyme does not correlate with the increased expression demonstrated here; this may suggest a blockade of activity caused by oxidative damage to the enzyme structure [20].

Numerous studies have demonstrated that neuronal damage, such as that which occurs in Alzheimer's disease (AD), increases the expression and activation of cell cycle regulatory proteins [21]. It has been shown that D-type cyclins play an important role in memory, learning, and neuronal plasticity processes [22]. These processes are linked to the formation of new neurons from neuroblasts in the DG of the hippocampus [23]. However, the expression of these proteins in mature neurons activates death pathways in the cell [24]. The damage generated by chronic exposure to low doses of ozone induced an increase in the expression of cyclin D2 in the hippocampi of the treated animals (Figure 3). The results show an increase in the expression of this protein beginning at 7 days and continuing throughout all treatments; however, cyclin D2 only translocates into the nucleus after 30 days of exposure to ozone. This translocation may be associated with the increase in active caspase 3 at 30 and 60 days of exposure to ozone (Figure 5), suggesting the activation of apoptotic pathways. The increase in cyclin D2 after short periods of ozone exposure (7, 15, and 30 days) could be associated with the neurogenesis process. We previously reported that, under the same conditions, the number of neuroblasts increases from 7 to 30 days of treatment; however, after 30 days of exposure to ozone, there is an increase in the death of neuroblasts and therefore a loss of neuronal repair processes [18]. Under normal conditions, mature neurons inhibit signals triggering cell cycle reentry through the phosphorylation of FoxO 1a (pFoxO 1a) because this transcription factor increases the expression of p27, which blocks the synthesis of cyclin D2 [16]. Thus, FoxO 1a functions as a tumor suppressor [25]. The present results show a significant increase in pFoxO 1a at 15, 30, and 60 days of exposure to ozone; however, this increase does not correlate with its repressive effect on cyclin D2.

In addition to the increased cyclin D2 in the nucleus and the increased expression of active caspase 3, which were demonstrated in this work, we previously reported increased translocation of p53 to the nucleus, which is a signal that activates apoptotic death in mature neurons [18]. Many authors have reported that both p53 and FoxO 1a transcription factors are involved in the same signaling pathways related to survival and cell death. These pathways are well regulated under redox balance; however, the loss of this balance alters these pathways. After DNA damage, p53 could inhibit the action of FoxO 1a [26]. Thus, the loss of redox balance increases cell death, likely by activating many pathways that induce apoptosis when mature neurons suffer cell damage. Similar phenomena could occur in neurodegenerative diseases associated with a chronic loss of redox balance.

**Figure 6:**
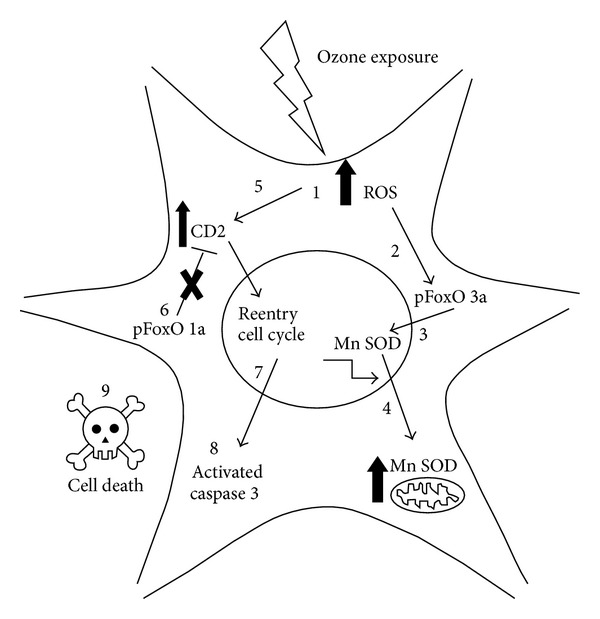
(1) We have shown previously that exposure to low doses of ozone increases the amount of reactive oxygen species (ROS) in neurons. (2) Our results demonstrate that an increase in ROS promotes FoxO 3a phosphorylation and its translocation to nucleus. (3) Once translocated, FoxO 3a increases the transcription of Mn SOD. (4) Mn SOD levels increase in the mitochondria. (5) We suggest that ROS damage increases the expression of cyclin D2. (6) Under redox balance, FoxO 1a is able to block cyclin D2; however, oxidative stress could inhibit the activity of FoxO 1a. (7) Thus, increased cyclin D2 promotes cell cycle reentry. (8) Because mature neurons cannot divide, cell death pathways are activated, including the activation of caspase 3. (9) Catastrophic apoptosis could be induced.

In summary, the exposure to low doses of ozone induced an increase in the expression of FoxO 1a, FoxO 3a, Mn SOD, cyclin D2, and caspase 3, from 7 to 60 days of exposure; the highest increase was observed at day 30 of exposure, while at 60 and 90 days the response showed a tendency to decrease. This behavior could be the result of the loss in the antioxidant systems regulation and/or an increase in neuronal death by apoptosis. Activation of FoxO 3a is associated with an increase in the expression of Mn SOD; however, this increase is unrelated to an increase in Mn SOD activity. On the other hand, ozone also increases the expression and nuclear translocation of cyclin D2, thereby activating the cell cycle, which triggers death signals in mature neurons. This process may be associated with increased expression of active caspase 3. Under normal redox balance, increased expression of cyclin D2 could be blocked indirectly by activation of FoxO 1a; however, an imbalance in the redox state could modify this response (Figure 6).

## 5. Conclusions

We conclude that the oxidative stress generated by chronic exposure to low doses of ozone alters regulatory pathways that attempt to counteract the production of ROS via upregulation of Mn SOD; however, ozone exposure does not affect the activity of these enzymes. On the other hand, the loss of redox balance causes alterations in intracellular signaling pathways, increases oxidative damage, and consequently induces the aberrant cell cycle reentry of mature neurons, which ends in the induction of catastrophic apoptosis. The failure of these protection mechanisms, together with neurodegeneration processes, generates a vicious cycle of degeneration mechanisms.
